# Photodynamic therapy in the treatment of basal cell carcinoma

**Published:** 2013-03-25

**Authors:** C Matei, M Tampa, T Poteca, G Panea-Paunica, SR Georgescu, RM Ion, SM Popescu, C Giurcaneanu

**Affiliations:** * “Carol Davila" University of Medicine and Pharmacy, Department of Oncologic Dermatology and Allergology, “Elias" University Emergency Hospital, Bucharest; ** “Carol Davila" University of Medicine and Pharmacy, Department of Dermatology, Faculty of Dental Medicine, “Victor Babes" Hospital, Bucharest; *** “Carol Davila" University of Medicine and Pharmacy, Department of General Surgery, “Colentina" Hospital, Bucharest; **** General Surgery Clinic, “Sf. Pantelimon" Clinical Emergency Hospital, Bucharest; ***** The National Institute for Research and Development in Chemistry, Bucharest; ****** IInd Dermatology Clinic, Colentina Hospital, Bucharest

**Keywords:** Photodynamic therapy, basal cell carcinoma, aminolevulinic acid, methyl aminolevulinate

## Abstract

Photodynamic therapy (PDT) is a medical procedure based on the activation of the molecules of various exogenous or endogenous chemical substances called photosensitizers by a light source emitting radiation of an adequate wavelength, usually situated in the visible spectrum; photosensitizers are chemical compounds bearing the capacity to selectively concentrate in the neoplastic cells. The energy captured by the molecules of these substances pervaded in the tumor cells is subsequently discharged in the surrounding tissue, triggering certain photodynamic reactions that result in the destruction of the tumor. The procedure is applicable in numerous medical fields. Skin basal cell carcinoma (BCC), the most frequent type of cancer of the human species, is a cutaneous tumor that responds very well to this innovative treatment method. By reviewing numerous recent studies in the field, this article aims to present the role and the indications of photodynamic therapy in the management of basal cell carcinoma, as well as the most important results achieved so far by this therapy in the field of dermato-oncology.

## Introduction

Basal cell carcinoma (BCC) is a malignant tumor arising from basal cells situated in the external root sheath of the hair follicle [**1,2**]. BCC is the most common type of cancer in humans, with over 1 million new cases occurring yearly in the United States and 53.000 new cases registered annually in the United Kingdom [**3**]. The basal cell carcinoma/squamous cell carcinoma (BCC/SCC) ratio varies widely in the medical literature, with most of the data indicating a 3-4 fold higher incidence for BCC, as compared to that of SCC [**4**]. UV radiation, skin aging, exposure to ionizing radiation and repeated mechanical trauma are involved, inter alia, in the development of cutaneous BCC. Genetic factors play a role of paramount importance, as individuals with low Fitzpatrick phototype present a higher risk of developing BCC. There are also genetic diseases characterized by a higher propensity towards developing basal cell carcinomas, i.e. Gorlin-Goltz syndrome and xeroderma pigmentosum. There are several different clinical types of the BCC: nodular, superficial, cystic, sclerosing (morpheaform) and pigmented BCC, the first two of which being the most frequently encountered in medical practice [**4,5**].

BCC treatment must be individualized based on considerations of the type and location of the tumor, the age of the patient and the associated comorbidities. As treatment alternatives, one could turn to surgical excision, Mohs microsurgery, curettage with or without electrosurgery, cryosurgery, topical cytostatic drug preparations (5-fluorouracil 5%, imiquimod 5%) and radiotherapy [**1,3**]. Photodynamic therapy (PDT) is a procedure that has only recently become accepted for the treatment of BCC; its utility in the treatment of this particular skin tumor will be discussed as it follows.

**Photodynamic therapy **is a medical procedure based on the activation of the molecules of various exogenous or endogenous chemical substances called photosensitizers by a light source emitting radiation of an adequate wavelength, usually situated in the visible spectrum; the photosensitizers are chemical compounds with the property of being able to selectively concentrate in the neoplastic cells. The energy captured by the molecules of the substances captured within the tumor cells is subsequently discharged in the surrounding tissue, triggering certain photodynamic reactions that result in the destruction of the tumor [**6**].

 Photodynamic therapy requires the simultaneous presence of a light source, a photosensitizer and tisular oxygen. The light sources used in photodynamic therapy may be either incoherent polychromatic sources (gas discharge lamps, LEDs) or coherent monochromatic sources (lasers). With respect to the photosensitizers, most of the studies conducted up to this moment concentrate on the use of porphyrin precursors, especially aminolevulinic acid (ALA) and its methylated ester, methyl aminolevulinate (MAL).

 The porphyrin precursors (ALA and esters of ALA) are topically administered and have a high capacity of cutaneous penetration. ALA crosses the cell membranes and reaches the interior of the skin cells without any chemical change, while ALA esters (such as MAL) are hydrolyzed in the cytoplasm of the tumor cells, releasing ALA in the cytosol. Subsequently, ALA will be taken over by the enzymatic equipment of the heme synthesis, readily available in the skin cells, and is transformed in protoporphyrin IX, a substance with an important photosensitizing activity, that will accumulate intracellularly - this being the actual target molecule of photodynamic therapy. After protoporphyrin IX molecule absorbs a quantum of energy provided by the light source used in PDT, the molecular electronic transitions lead to photodynamic reactions, giving birth to a certain number of reactive oxygen species: superoxide anion (O2-), hydroxyl radical (OH•) and singlet oxygen (1O2), the latter being extremely aggressive and considered the main promoter of destructive cellular effects exerted by PDT [**6**]. In the tumoral cells, photodynamic therapy can induce either autophagy or cellular necrosis- in case of a great intensity oxidative stress- as well as apoptosis, programmed cell death- in case of a moderate intensity oxidative stress [6,7].

**Photodynamic therapy with aminolevulinic acid (ALA-PDT) in the treatment of BCC**

 A retrospective study published by Christensen et al., performed on 44 patients with 60 histopathologically confirmed basal cell carcinoma lesions, treated 6 years beforehand with ALA-PDT (ALA in DMSO, 1-2 sessions), revealed complete remission of the lesions in 81% of the cases (68% in the ones on which a single session of ALA-PDT was performed and 91% in the ones on which two sessions were performed). 9 out of 10 patients graded the results of the procedure as very satisfying [**8**]. A subsequent study published by the same authors evaluated the clinical and histological changes at 10 years after photodynamic therapy with topical ALA and assessed a rate of complete remission of 60% in the cases on which only one single session of ALA-PDT has been performed and 87% after two therapy sessions. The aesthetic result after 10 years was considered as being very good in over 90% of the patients included in the study [**9**].

 Thissen et al. showed in a study published in 2000 that, out of 24 basal cell epithelioma lesions treated with ALA-PDT at a fluence rate of 100mW/cm2 and irradiance of 120J/cm2 at 3 weeks after curettage, 22 lesions completely remitted; the remission was confirmed both clinically and histopathologically [**10**].

 A study published by Souza et al. in 2009 followed the effects of ALA-PDT on a group of patients presenting 19 plaques of Bowen disease and 15 basal cell epitheliomas, on which topical ALA (20% w/v, combined with DMSO and EDTA) was applied under occlusion for 6 hours. After the irradiation with red light of 630 nm provided by a diode laser at fluence rates of 100-300J/cm2, complete remission at 3 months after therapy was achieved in over 90% of the treated lesions; the remission maintained at 5 years follow-up in approximately 60% of the treated cases [**11**].

 Wang et al. showed, in a third phase study conducted on 88 patients with basal cell epitheliomas, that the efficiency of ALA-PDT is similar to that of cryosurgery, but, compared to the later, it has the advantage of a greater cosmetic result, achieved in a lesser healing interval [**12**].

 A study published in 2012 by Osiecka and col. evaluates the use of combined therapy encompassing both ALA-PDT and imiquimod 5% on a group of 34 patients presenting histopathologically confirmed BCC lesions; the patients were randomized between ALA-PDT combined with vehicle placebo cream (n=10 patients) and ALA-PDT combined with topical applications of imiquimod (n=24 patients); the authors encountered a complete remission in 60% of the patients from the first group, compared to 75% in the second group. These results led to the conclusion that ALA-PDT combined with imiquimod is a useful therapeutic alternative in BCC treatment; moreover, in the group treated with the above-mentioned combined therapy, the aesthetic results were excellent [**13**].

 Nevertheless, ALA-PDT does not seem to be an efficient option for the treatment of nodular forms of basalioma. A controlled randomized study published in 2008, which enrolled 149 patients with 173 BCCs, 88 of which were surgically excised with safety margins of 3 mm and the rest of 85 lesions were treated with PDT at 4 hours after applying ALA, proved a recurrence rate at 3 years post-therapy of 2.3% for the surgical excision, compared to 30.3% for PDT [**14**].

 Patients suffering from nevoid basal cell carcinoma syndrome (Gorlin-Goltz syndrome) can also benefit from this procedure. Itkin and Gilchrest proved in a study published in 2004 that photodynamic therapy with topical ALA 20% and activated by blue light (λ=417 nm) at a fluence rate of 10 mW/cm2 for a period of 1000 seconds is efficient in the management of Gorlin-Goltz syndrome. ALA was applied within up to 5 hours before illumination with PDT light source, and the sessions were repeated at 2-4 months intervals; complete remissions were observed in 89% of the cases of superficial BCC and 31% of the cases of nodular BCC treated, without any recurrence encountered over a period of eight months post-therapeutic follow-up [**15**].

 Chapas and Gilchrest reported excellent results after using blue light (λ=410 nm, fluence rate of 10J/cm2) ALA-PDT in 4 sessions repeated at a period of 2-3 months in a patient with Gorlin-Goltz syndrome, observing not only the remission of the treated lesions, but also the significant improvement of the aesthetic appearance of the preexisting scars and the decrease in the rate of new lesions occurrence [**16**]. Other studies also present ALA-PDT as an efficient therapy for cutaneous lesions in Gorlin-Goltz syndrome, with very good aesthetic results [**17**].

** Photodynamic therapy with methyl aminolevulinate (MAL-PDT) in the treatment of BCC**

 MAL-PDT is a very safe and efficient therapeutic approach in the treatment of basal cell carcinomas. In 2001, Soler et al. published a study on 350 superficial BCCs and previously curetted nodular BCCs, on which 160 mg/g concentration MAL was applied, at 3 hours before illumination with a PDT lamp. The light source used in the study was a wide spectrum halogen lamp, emitting at a fluence rate of 50-200J/cm2. At the end of the study, 310 of the 350 treated lesions remitted completely, 89% of them maintaining the remission even after 3 years after post-therapeutical follow-up. The cosmetic results were reported as good or excellent in 98% of the cases that achieved complete remission [**18**]. 

 A study published in 2011 evaluated the efficiency of MAL-PDT in 135 patients with 194 BCCs; the complete response rate was of 62% in the whole studied group, with a stratification of 82% for superficial BCCs and 33% for nodular BCCs. The study proved that both limb location of BCCs and the presence of ulceration negatively affect the efficacy of MAL-PDT. The size of the lesions does not seem to influence the outcome of the procedure [**19**].

 Moreover, it was observed that the thickening of the lesion parallels the decrease in the efficacy of the procedure, a phenomenon most likely related to the physical ability of the utilized luminous radiation to penetrate the skin. A study conducted on 47 patients presenting 95 BCCs of different thickness on whom red light of 633 nm MAL-PDT at a fluence rate of 339J/cm3 was performed, stratified the results depending on the thickness of the lesion, as it follows: complete response- in lesions with thickness less than 1.3 m, partial response-in lesions between 1.3 and 1.8 mm and lack of response/nonresponsive- the ones that exceeded 1.8 mm in thickness. The total response rate was of 75.8% at 24 months after the procedure and the cosmetic results were rated as satisfactory by 90.3% of the treated patients [**20**].

 Two randomized, multicentre, double-blind trials performed in Australia and published in 2009 plead for the efficiency of MAL-PDT in the treatment of nodular BCC. The trials enrolled 131 patients, of which 66, presenting 75 epitheliomas, were referred for MAL-PDT treatment, and 65 patients presenting 75 epitheliomas were treated with placebo-PDT. After a slight debridation and surgical reduction of the tumor size, the irradiation was performed by using an incoherent red light source (570-670 nm, 75 J/cm2), and the sessions were repeated at one week interval; of all treated lesions, the ones that were only partially responsive at 3 months post-therapy were subjected to another treatment session. The treated cutaneous areas were subsequently excised and examined histopathologically; a complete remission rate of 75% was observed in the patient treated with MAL-PDT, as compared to only 27% in the group treated with placebo-PDT. Better results were obtained after the treatment of BCCs that were situated on the face -89% complete remission rate. The aesthetic response was considered good or very good in 98% of the cases with complete remission [**21**].

 MAL-PDT is also efficient in the therapeutical management of BCCs that are considered “difficult to treat" –in respect to either their dimension or topographic location (periorificial, nasal region, auricular pavilion, etc) or the potential for developing surgical complications- as in patients who follow an anticoagulant treatment and/or present a significant cardiovascular risk. An open, prospective, uncontrolled study assessed 94 patients with 123 “difficult to treat" basal cell epitheliomas, submitted in 9 European referral centers to 1 or 2 therapeutic cycles on a period of 3 months, each cycle consisting in 2 sessions of MAL-PDT separated by a one week interval. 9 patients (totalizing 15 lesions) were excluded from the study by an independent committee, since they were considered not to fulfill the conditions for “difficult to treat" BCCs. The remission rates 3 months after completion of the therapy were 92% for superficial BCC and 87% for the nodular ones. Recurrences were registered in 18% of the treated cases after 2 years, data at which the aesthetic results were still very good in about 94% of the cases [**22**]. 

 In 2009, Togsverd-Bo et al. showed that MAL-PDT is an efficient method for the treatment of basal cell epitheliomas of the eyelid margin, and described a therapeutic protocol that guarantees the safety of the therapy in ophthalmology [**23**]. A series of 6 patients with BCCs of the eyelid margin- one of whom presenting a superficial BCC and 5 of whom presenting nodular BCCs were subjected to MAL-PDT in two sessions at one week interval and achieved complete remission that maintained for over 2 years; nonetheless, more extensive studies in order to establish the safety and efficacy of MAL-PDT treatment in the management of the eyelid epitheliomas are necessary [**24**].

 MAL-PDT also proves to be very efficient in treating giant basaliomas. In 2008, Eibenschutz et al. presented a series of cases of giant (14 lesions larger than 50 mm in diameter) and large (5 lesions with a diameter between 40 and 49 mm) basal cell epitheliomas treated with MAL-PDT, showing 95% remission rate after 6 months; 3 years post-therapy, the remission rate was maintained in 66% of the treated cases [**25**]. In the case of giant BCCs, the association of MAL-PDT and topical imiquimod, 5% treatment leads to a considerable reduction in the tumoral dimensions, allowing a subsequent excision to be performed in safe conditions; it was reported that, in the case of three patients, 3 cicles of MAL-PDT followed by a 6 week treatment with topical imiquimod 5% determined the reduction of giant BCCs to dimensions that allowed a good surgical excision, considerably decreasing the risk of intra and post-operative complications [**26**].

 MAL-PDT is also efficient in patients suffering from nevoid basal cell carcinoma syndrome of Gorlin-Goltz; a study performed on a series of 7 patients showed a high satisfaction degree in 85% of the patients treated with MAL-PDT, compared to only 55% in the patients treated with repeated surgical excisions [**27**]. Fibroepithelioma of Pinkus, an uncommon form of BCC, seems to respond poorly to MAL-PDT, even after up to 9 repeated sessions; extensive data obtained from larger trials are lacking, so far only scattered data concerning the treatment of this particular form of BCC being available in the medical literature [**28**].

 The efficiency of MAL-PDT is comparable to that of cryosurgery, but the cosmetic outcome is better. A multicenter clinical trial published in 2008 compared the efficiency of MAL-PDT with that of cryotherapy in the treatment of superficial BCCs. 60 patients with 114 lesions and 58 patients with 105 lesions were randomized between the two therapies; red light (570-670 nm) PDT was performed on the patients in the first group at 3 hours after applying MAL under occlusion, while the patients in the second group were subjected to cryosurgery. MAL-PDT was repeated after 3 months in 20 patients who did not obtain remission (two sessions separated by a one week period), and so was cryosurgery for 16 similar patients. Complete remission was obtained in case of 100 lesions treated with PDT and 93 lesions treated with cryosurgery. The recurrence rates after 5 years were similar (22% for MAL-PDT and 20% for cryosurgery), but the aesthetic results were better in the patients treated with MAL-PDT (60% of the patients in the first group and 16% in the second group rated the cosmetic results as excellent) [**29**].

A prospective multicenter study performed on 101 patients with nodular BCCs, randomized between two sessions of MAL-PDT using red light (570-670 nm, 75 J/cm2), separated by a 7 day interval (n=52 patients) and surgical excision (n=49 patients), proved similar response rates after 3 months (91% and 98% respectively), an outcome that was maintained after 1 year in 83% of the patients treated with PDT, as compared to 96% for the surgical treatment. Yet, the cosmetic result was appreciated by both the dermatologist and the patient as being better after MAL-PDT, than after the surgical excision [**30**]. Moreover, a study performed on 196 patients with a total number of 274 lesions (an average of 1.4 BCCs per patient) showed a reduction of 92.2% in the number of lesions at 3 months after MAL-PDT, compared to 99.2% after the surgical excision; after 12 months, the recurrence among patients treated with MAL-PDT was of 9.3%, while in the group subjected to surgery no lesion recurrence was observed. Nevertheless, the aesthetic result, as appreciated by the investigators after 12 months post-therapy was good or excellent in 94.1% of the patients treated with MAL-PDT, compared to only 59.8% in those subjected to surgical intervention [**31**]. 

## Conclusions

Summarizing the presented data, we can assert that MAL-PDT is a useful method for the treatment of basal cell epitheliomas, more efficient than placebo-PDT and with a similar efficiency to cryosurgery. Even though the recurrence rate of the lesions is higher than after surgical excision, the excellent aesthetic results strongly recommend it, especially since it does not impede or harden a subsequent reintervention, such as PDT or surgical excision. Moreover, the procedure has good results in approaching the lesions that are located in areas that are difficult to treat by other methods and in patients in whom, because of various medical reasons, the surgical intervention is contraindicated. The procedure can also be employed in the treatment of patients suffering from Gorlin-Goltz syndrome, where the high number of basal cell carcinomas makes other therapeutical approaches less suitable. In the case of epitheliomas that are non-responsive to MAL-PDT, increasing the number of sessions does not bring any considerable improvement. The thickness of the tumor is an important prediction factor for the effectiveness of the procedure, as superficial lesions respond much better to PDT than nodular ones. Further identification of new photosensitizers and new methods of increasing their penetrance in the tumors, as well as the design of new therapeutic protocols could become -in the near future- the foundation of enhancing PDT efficiency in the treatment of basal cell carcinomas.

## References

[1] Sterry  W, Paus  R (2006). Dermatology.

[2] Lang  PG, McKelvey  AC (1987). Three-dimensional reconstruction of the superficial multicentric basal cell carcinoma using serial sections and a computer. Am J Dermatopathol.

[3] Smith  V, Walton  S (2011). Treatment of facial Basal cell carcinoma: a review. J Skin Cancer.

[4] Carucci  JA, Leffell  DJ (2008). Basal cell carcinoma. In: Wolff K, Goldsmith LA, Katz SI, et al. eds.: Fitzpatrick's Dermatology in General Medicine, 7th ed.

[5] Castori  M, Morrone  A (2003). Genetic skin diseases predisposing to basal cell carcinoma. Eur J Dermatol.

[6] Ion RM (2003). Porfirinele si terapia fotodinamica a cancerului.

[7] Kessel  D (2012). Subcellular targets for photodynamic therapy: implications for initiation of apoptosis and autophagy. J Natl Compr Canc Netw.

[8] Christensen , Skogvoll  (2009). Photodynamic therapy with 5-aminolaevulinic acid, dimethylsulfoxide and curettage in basal cell carcinoma: a 6-year clinical and histological follow-up. J Eur Acad Dermatol Venereol.

[9] Christensen  E, Mork  C (2012). High and sustained efficacy after two sessions of topical 5-aminolaevulinic acid photodynamic therapy for basal cell carcinoma: a prospective, clinical and histological 10-year follow-up study. Br J Dermatol.

[10] Thissen  MR, Schroeter  CA (2000). Photodynamic therapy with delta-aminolaevulinic acid for nodular basal cell carcinomas using a prior debulking technique. Br J Dermatol.

[11] Souza CS, Felicio  LB (2009). Long-term follow-up of topical 5-aminolaevulinic acid photodynamic therapy diode laser single session for non-melanoma skin cancer. Photodiagnosis Photodyn Ther.

[12] Wang I, Bendsoe  N (2001). . Photodynamic therapy vs. cryosurgery of basal cell carcinomas: results of a phase III clinical trial. Br J Dermatol.

[13] Osiecka  B, Jurczyszyn  K (2012). The application of Levulan-based photodynamic therapy with imiquimod in the treatment of recurrent basal cell carcinoma. Med Sci Monit.

[14] Mosterd  K, Thissen  MR (2008). Fractionated 5-aminolaevulinic acid-photodynamic therapy vs. surgical excision in the treatment of nodular basal cell carcinoma: results of a randomized controlled trial. Br J Dermatol.

[15] Itkin  A, Gilchrest  BA (2004). delta-Aminolevulinic acid and blue light photodynamic therapy for treatment of multiple basal cell carcinomas in two patients with nevoid basal cell carcinoma syndrome. Dermatol Surg.

[16] Chapas  AM, Gilchrest  BA (2006). Broad area photodynamic therapy for treatment of multiple basal cell carcinomas in a patient with nevoid basal cell carcinoma syndrome. J Drugs Dermatol.

[17] Gilchrest  BA, Brightman  LA (2009). Photodynamic therapy for patients with Basal cell nevus syndrome. Dermatol Surg.

[18] Soler  AM, Warloe  T (2001). A follow-up study of recurrence and cosmesis in completely responding superficial and nodular basal cell carcinomas treated with methyl 5-aminolaevulinate-based photodynamic therapy alone and with prior curettage. Br J Dermatol.

[19] Fantini  F, Greco  A (2011). Photodynamic therapy for basal cell carcinoma: clinical and pathological determinants of response. J Eur Acad Dermatol Venereol.

[20] Li Q, Gao  T (2010). Clearance of a thick invasive squamous cell carcinoma after multiple treatments with topical photodynamic therapy. Photomed Laser Surg.

[21] Foley  P, Freeman  M (2009). Photodynamic therapy with methyl aminolevulinate for primary nodular basal cell carcinoma: results of two randomized studies. Int J Dermatol.

[22] Horn  M, Wolf  P (2003). Topical methyl aminolaevulinate photodynamic therapy in patients with basal cell carcinoma prone to complications and poor cosmetic outcome with conventional treatment. Br J Dermatol.

[23] Togsverd-Bo  K, Haedersdal  M (2009). Photodynamic therapy for tumors on the eyelid margins. Arch Dermatol.

[24] Kotimaki  J (2009). Photodynamic therapy of eyelid basal cell carcinoma. J Eur Acad Dermatol Venereol.

[25] Eibenschutz  L, Marenda  S (2008). Giant and large basal cell carcinoma treated with topical photodynamic therapy. Eur J Dermatol.

[26] Madan  V, Loncaster  JA (2006). Nodular basal cell carcinoma in Gorlin's syndrome treated with systemic photodynamic therapy and interstitial optical fiber diffuser laser. J Am Acad Dermatol.

[27] Pauwels  C, Mazereeuw-Hautier  J (2011). Topical methyl aminolevulinate photodynamic therapy for management of basal cell carcinomas in patients with basal cell nevus syndrome improves patient's satisfaction and reduces the need for surgical procedures. J Eur Acad Dermatol Venereol.

[28] Park  MY, Kim  YC (2010). Fibroepithelioma of Pinkus: poor response to topical photodynamic therapy. Eur J Dermatol.

[29] Basset-Seguin  N, Ibbotson SH (2008). Topical methyl aminolaevulinate photodynamic therapy versus cryotherapy for superficial basal cell carcinoma: a 5 year randomized trial. Eur J Dermatol.

[30] Rhodes  LE, De Rie  M (2004). Photodynamic therapy using topical methyl aminolevulinate vs surgery for nodular basal cell carcinoma: results of a multicenter randomized prospective trial. Arch Dermatol.

[31] Szeimies  RM, Ibbotson  S (2008). A clinical study comparing methyl aminolevulinate photodynamic therapy and surgery in small superficial basal cell carcinoma (8-20 mm), with a 12-month follow-up. J Eur Acad Dermatol Venereol.

